# Microbiological contamination of young children’s hands in rural Bangladesh: Associations with child age and observed hand cleanliness as proxy

**DOI:** 10.1371/journal.pone.0222355

**Published:** 2019-09-10

**Authors:** Sarker Masud Parvez, Rashidul Azad, Amy J. Pickering, Laura H. Kwong, Benjamin F. Arnold, Musarrat Jabeen Rahman, Md. Zahidur Rahman, Mahfuja Alam, Debashis Sen, Sharmin Islam, Mahbubur Rahman, John M. Colford, Stephen P. Luby, Leanne Unicomb, Ayse Ercumen

**Affiliations:** 1 International Centre for Diarrhoeal Disease Research, Bangladesh (icddr,b), Dhaka, Bangladesh; 2 School of Engineering, Tufts University, Medford, MA, United States of America; 3 Stanford University, Stanford, CA, United States of America; 4 Division of Epidemiology and Biostatistics, School of Public Health, University of California, Berkeley, CA, United States of America; 5 North Carolina State University, Raleigh, NC, United States of America; University of Auckland, NEW ZEALAND

## Abstract

**Background:**

Hands are a route of transmission for fecal-oral pathogens. This analysis aimed to assess associations between hand *E*. *coli* contamination and child age and determine if observed hand cleanliness can serve as a proxy for *E*. *coli* contamination on young children’s hands.

**Methods:**

Trained field workers collected hand rinse samples from children aged 1–14 months in 584 households in rural Bangladesh and assessed the visual cleanliness of child hands (fingernails, finger pads and palms). Samples were analyzed using the IDEXX most probable number (MPN) methodto enumerate *E*. *coli*. We assessed if child age (immobile children aged 1–4 months vs. mobile children aged 5–14 months) is associated with log_10_ *E*. *coli* counts on hands using generalized estimating equations (GEE). We estimated the log_10_ difference in hand *E*. *coli* counts associated with the cleanliness of different hand parts using a multivariable GEE model.We calculated the sensitivity, specificity, positive predictive value (PPV) and negative predictive value (NPV) for dirty fingernails, fingerpads, palms and overall hands (the three observed parts combined) against binary *E*. *coli* presence on hands.

**Results:**

*E*. *coli* was detected on 43% of child hands. Children in the mobile age range had 0.17 log_10_ MPN higher *E*. *coli* on hands than those in the immobile age range (Δlog_10_ = 0.17, 95% CI = 0.02, 0.32, *p* = 0.03). Children with visible dirt particles on finger pads had 0.46 log_10_ MPN higher *E*. *coli* on hands than those with clean finger pads (Δlog_10_ = 0.46, 95% CI = 0.05, 0.87, *p* = 0.03). Dirty fingernails indicated binary *E*. *coli* presence with 81% sensitivity and 26% specificity while dirty fingerpads and palms indicated *E*. *coli* presence with 29% sensitivity and 75–77% specificity. The PPV was 45–48% and NPV 59–65% for all three types of observations.

**Conclusion:**

Hand contamination with *E*. *coli* was prevalent among young children in rural Bangladesh, with higher levels of contamination among mobile children. Studies should assess if strategies to remove animal feces from the courtyard, provide designated hygienic play spaces for children and deliver targeted messaging to mothers to wipe or wash children’s hands after contact with animals and animal feces reduce child hand contamination. Visible hand cleanliness was a poor predictor of *E*. *coli* presence on young children’s hands so other low-cost field measurements are needed to accurately detect fecal contamination on hands.

## Background

Fecal-oral pathogens are transmitted through multiple environmentally mediated pathways such as water, food, hands, fomites, and flies [1, 2]. Hands are an important route for pathogen transmission [3]. Fecal indicator bacteria and pathogens, including rotavirus, norovirus, *Shigella* spp., and *Cryptosporidium* spp. have been detected on hands. Soil-transmitted helminth ova and larvae have also been detected under fingernails [4–7]. Pathogens can survive for several hours on hands; for example, rotavirus can survive for over 4 hours on the hand surface [8]. In rural low-income country settings, young children frequently touch soil, play with domestic animals or near cowsheds and come into contact with animal feces, presenting ample opportunities for hand contact with sources of contamination with fecal pathogens [9]. Previous studies in rural Bangladesh have shown that, 72% of households have earthen floors, 94% have domestic poultry and livestock, and 89% have animal feces observed within the compound [10–12]. Human and animal fecal markers have been detected on child hands in Bangladesh [13] and India [5].

Hand contamination poses both direct and indirect risks for pathogen transmission. Direct exposure results from hand-to-mouth contacts while indirect risks result from ingestion of food and drinking water that have been contaminated by hands [14]. Young children often put their hands into their mouth [9], and a single contact can transfer 33–41% of microbiological contamination from contaminated hands tothe mouth [15]. In rural Bangladesh, children typically eat by hand and could also ingest pathogens from contaminated hands via food. Two systematic reviews and meta-analyses reported that effective hand hygiene interventions reduce gastrointestinal illness by 23–31% and respiratory infection by 21% [16, 17].

Assessment of fecal contamination on hands may help us understand the role of hands in fecal-oral pathogen transmission and evaluate the effectiveness of hand hygiene programs. There are several approaches to assess hand cleanliness. Testing for microbiological contamination requires laboratory facilities and trained personnel [18]. Visual spot checks of hand cleanliness could be a proxy for microbiological hand contamination and provide asimple and rapid means to assess hand cleanliness in field studies. Visual hand cleanliness among caregivers and children has been shown to be associated with water availability at handwashing locations in rural Bangladesh, suggesting it could be a proxy for handwashing practices [19]. Observed soil on farmers’ hands was correlated with microbial hand contamination as measured by *Enterococcus* but not *E*. *coli* in Mexico [20]. In Tanzania, visible hand contamination was associated with fecal *streptococci* and *E*. *coli* measured on hands of caregivers and children aged <5 years [4].

Among young children, there could be heterogeneity in levels of hand contamination and in the association between observed hand cleanliness and *E*. *coli* presence by age groups associated with different motor milestones. In a recent study in Bangladesh, structured observations among children aged 3–18 months found varying hand-mouthing frequency with age [9]. Children who are immobile (0–4 months) may also have different patterns of hand-environment contacts than those crawling and learning to walk (5–18 months) as children who can pick up objects and move to different parts of the compound may have more contact with human and animal fecal sources in the compound environment [21]. Soil in the compound courtyard,where young children spend a lot of time in rural Bangladeshi households, containshigh concentrations of *E*. *coli* [12]. *E*. *coli* can be naturally present in tropical soils and does not necessarily indicate fecal contamination [22, 23], and it also does not differentiate between human vs. animal fecal sources which differ in the health risk they pose [24]. Nonetheless, our previous work in Bangladesh showed an association between *E*. *coli* on child hands and diarrhea and bloody stools in children <5 years, suggesting fecal-oral pathogen transmission by contaminated child hands [25]. However, subgroup analyses showed that a relationship between *E*. *coli* on hands and diarrhea was observed among children 0–5 months old but not among children 6–23 or 24–60 months old, suggesting age-associated nuances [25].

In the analysis reported here, we focus on young children aged 1–14 months to (1) identify the prevalence and concentration of *E*. *coli* in child hand rinse samples, (2) assess associations between hand *E*. *coli* contamination and child age, and (3) determine how well observed hand cleanliness serves as a potential proxy for *E*. *coli* contamination of young children’s hands, with subgroup analyses by age group.

## Methods

### Study setting and population

This study was conducted in rural villages of four districts (Gazipur, Kishoreganj, Mymensingh, Tangail) in central Bangladesh. We enrolled households from among the participants of a large-scale randomized controlled trial of water, sanitation, hygiene and nutrition interventions in rural Bangladesh (WASH Benefits, NCT01590095). The study design and methods for the parent trial are described elsewhere [26]. The current study was nested within the control arm of WASH Benefits; we approached all households in one of the two groups in the double-sized control armof the trial to participate in this sub-study approximately one year after enrollment began in May 2012. WASH Benefits enrolled pregnant mothers with the objective of following their birth cohort (“index children”); as such the study population consisted of households with young children aged between 1–14 months at the time of our visit.

### Data collection and sample processing

Data collection was conducted between July 2013-March 2014 and covered both the dry season (Jul-Oct) and rainy season (Nov-Mar). Trained field workers administered a structured questionnaire, performed spot checks on hand cleanliness and domestic hygiene and collected child hand rinse samples. The questionnaire collected data on household socio-demographic factors, household assets and animal ownership. The spot check observations included sanitation and hygiene conditions in the compound, including the presence of a handwashing station near the kitchen and latrine, improved latrine, and human and animal feces in the compound.

Child hand observations and hand rinses were collected from index children, and if not available, from the next youngest child in the compound. A field worker assigned to the household observed child hands including the fingernails, finger pads and palms of both hands for visible dirt using pre-specified appearance codes on a three-point scale, following classroom training by study investigators and subsequent field testing to standardize the method and ensure interrater reliability. If specks of visible dirt, mud, soil, ash or any other materials were visible on the specific part of the hand, it was coded as “visible dirt particles”. If there were no visible dirt particles on the observed part of the hand but it was generally unclean in color or appearance, it was coded as “unclean appearance”. If the observed part of the hand was clean as would appear after handwashing, it was coded as “clean”.

Child hand rinse samples were taken after the child hand observation. Field workers sampled child hands by rinsing both hands, one at a time, in a single Whirlpak bag (Nasco Modesto, Salida, CA) pre-filled with 250 mL of distilled water. Each hand was massaged from the outside of the bag for 15 seconds, followed by 15 seconds of shaking, and the rinse water was preserved in the Whirlpak bag [27]. Samples were preserved on ice and transported to the field laboratory to be processed on the same day, typically within 12 hours of collection. Upon arrival at the laboratory, samples were kept on ice until they were processed. Hand rinse samples were diluted 1:2 with distilled water (50 mL of sample diluted with 50 mL of distilled water). Samples were analyzed using the IDEXX Quanti-Tray 2000 system with Colilert-18 media (IDEXX Laboratories, Inc., Westbrook, ME) and incubated for 18 hours at 44.5° C to enumerate the most probable number (MPN) of *E*. *coli* [28]. The *E*. *coli* concentration was expressed as MPN per two hands. To accommodate variability in hand contamination, the Quanti-Tray 2000 system with a wide detection range (1−2419 MPN) per tray was selected. For quality control, field workers collected 10% field blank samples by opening and shaking a Whirlpak bag pre-filled with distilled water in the household as if collecting a hand rinse sample. Each laboratory technician processed one laboratory blank sample per day. In addition, 5% laboratory replicates (two aliquots from the same hand rinse sample) were processed. About10% of trays were re-counted by the lab supervisor to detect and minimize inter-counter variability. dx.doi.org/10.17504/protocols.io.2f9gbr6 [PROTOCOL DOI]

### Data analysis

We calculated log_10_-transformed *E*. *coli* counts, substituting the value of 0.5 MPN for samples below the detection limit or 2420 MPN for samples above the detection limit. We defined the binary outcome of *E*. *coli* presence/absence as the detection of ≥1 MPN *E*. *coli* per two hands. We categorized positive hand rinse samples into low, medium and high contamination strata (1–9, 10–99, and ≥100 MPN per two hands).

We estimated the log_10_ difference in hand *E*. *coli* counts associated with immobile (aged 1–4 months) vs. mobile (aged 5–14 months) children using generalized estimating equations (GEE) with a Gaussian error distribution and robust standard errors to account for clustering; the unit of clustering for this analysis was a group of eight neighboring households, defined by the WASH Benefits trial as a “cluster" [26] and used as the unit of assignment into study arms. Within each age group, we estimated the log_10_ difference in hand *E*. *coli* counts associated with each month increase in child age using GEE.

For each observed hand part (fingernails, fingerpads and palms), we compared log_10_-transformed hand *E*. *coli* concentrations between the three cleanliness categories with GEE, using indicator variables for visible dirt particles and unclean appearance against the reference category of clean. We also assessed associations between log_10_-transformed *E*. *coli* concentrations and the cleanliness of all different hand parts using a multivariable GEE model including indicator variables for visible dirt particles and unclean appearance for all hand parts.

To assess the predictive performance of observed hand cleanliness against binary *E*. *coli* presence, we collapsed the three-point hand observation scale into a binary indicator in two ways: (1) for our primary analysis, we coded visible dirt particles and unclean appearance as “dirty” (and otherwise as “clean”); (2) to conduct a sensitivity analysis to assess if our cleanliness definition affects our findings, we used an alternative definition where we coded hands with visible dirt particles as “dirty” (and otherwise as “clean”). Using these definitions, we generated indicator variables for dirty fingernails, dirty fingerpads and dirty palms if these were dirty on either hand and a composite indicator for dirty hands if any of the three was dirty on either hand. We calculated the sensitivity, specificity, positive predictive value (PPV) and negative predictive value (NPV) of these indicators, along with the corresponding 95% confidence intervals, against binary *E*. *coli* presence, both for all levels of *E*. *coli* contamination combined (**≥**1 MPN) and for the low, medium and high contamination strata defined above to assess if the performance of hand observations as a proxy varies with the level of *E*. *coli* contamination. We also calculated these diagnostic parameters for subgroups by child age (1–4 months vs. 5–14 months).

### Ethics

Respondents in the participating households (children's caregivers) provided written, informed consent before interviews and sample collection. The study protocol was reviewed and approved by Research Review Committee and Ethical Review Committee of the International Centre for Diarrhoeal Disease Research, Bangladesh (icddr,b) [PR#11063] and by the human subject committees of University of California, Berkeley [2011-09-3652] and Stanford University [25863].

## Results

### Enrollment

Among 699 households enrolled in the selected control arm of WASH Benefits, we successfully enrolled a total of 608 (87%) in our assessment, with 91 (13%) households not enrolled (7% stillbirth, miscarriage, abortion or index child death, 5% migration/absence, and 1% refusal. Among the 608 households enrolled into our assessment, we successfully collected child hand rinse samples from 584 (96%) households. The field team could not collect 24 (4%) hand rinse samples due to refusal.

### Demographics and water, sanitation and hygiene conditions

The mean age of mothers was 24 years and 85% of mother’s had formal education. The average age of the study children was 4.6 months, including an average of 1.3 children <5 years old per household. More than half (57%) of households had electricity while over 80% owned a mobile phone. The primary source of drinking water was a tubewell (98%). The majority of households (97%) had a latrine on the premises and 33% of households owned a private hygienic latrine (defined as presence of functional water seal and no visible feces on latrine slab/floor). Only 9% of children were reported to use a potty for defecation, and 10% of households reported disposing of child feces in a latrine. More than half (55%) of the households had a handwashing station within 10 meters of the latrine while only 11% had water and soap present at the handwashing station. Only 20% of households had a handwashing station near the kitchen and 12% had water and soap present at the station. Animal ownership was reported in 94% of household compounds, and 89% had animal feces observed within the compound. About 8% of respondents reported using an agricultural hoe to dispose of child feces and 35% to dispose of animal feces (Table 1).

**Table 1:** Demographic and household characteristics of study participants (N = 608).

Factors	Mean (SD)/n (%)
**Demographics**	
Mother’s age	23.8 (5.0)
Mother has some formal education(vs. none)	518 (85.2%)
Mean age of index children (in months)	4.6 (3.0)
Mean number of children <5 years in the household	1.3 (0.5)
**Socioeconomic indicators**	
Proportion who owned	
Electrical connection	349 (57.4%)
House	599 (98.6%)
Mobile phone	521 (85.7%)
Refrigerator	54 (8.9%)
House construction	
Tin roof	598 (98.4%)
Cement floor	71 (11.7%)
Brick walls	81 (13.3%)
Cooking fuel	
Wood	129 (21.2%)
Crop residue/grass	449 (73.9%)
Dung cakes	28 (4.6%)
Biogas	2 (0.4%)
**Water practices**	
Primary water source is tubewell	593 (97.6%)
**Sanitation practices**	
Access to on-site latrine	590 (97.1%)
Hygienic latrine^b^	188 (32.6%)
Child reported to defecate in potty	48 (7.9%)
Child feces reported to be disposed of in latrine	59 (9.7%)
Agricultural hoe present to remove feces	415 (68.3%)
Use of agricultural hoe reported to dispose of child feces	48 (7.9%)
Use of agricultural hoe reported to dispose of animal feces	211 (34.7%)
**Handwashing practices**	
Handwashing station within 10 meters of latrine	321 (55.7%)
Handwashing station within 10 meters of kitchen	123 (20.2%)
Water and soap in handwashing station near the latrine	66 (10.9%)
Water and soap in handwashing station near the kitchen	76 (12.5%)
Observed mother’s hand appears clean	104 (17.1%)
**Domestic hygiene**	
Reported animals in the compound^a^	572 (94.1%)
Observed animal feces in the compound^a^	539 (88.7%)
Observed animal roaming in the compound^a^	342 (59.9%)
Observed food waste in the courtyard	212 (34.9%)
Observed food waste where child recently spent time	130 (21.4%)
Observed wet soil in child play area	94 (15.5%)

SD: Standard deviation.

^a^Households in rural Bangladesh are clustered in multiple-family compounds (*baris*) shared by members of extended families.

^b^Hygienic latrine: functional water seal and no visible feces on the latrine slab/floor.

### *E*. *coli* on child hands

Out of 584 children,43% (n = 249) of child hand rinse samples contained *E*. *coli;* 31% had 1–9 MPN *E*. *coli*, 7% had 10–99 MPN *E*. *coli* and 5% had ≥100 MPN *E*. *coli*. The geometric mean *E*. *coli* concentration was 6.53 MPN per two hands. About 5% of samples exceeded the upper detection limit. Only 1% of blank samples were contaminated with *E*. *coli* and the intra-class correlation between samples processed in replicate was 86%.

### *E*. *coli* on hands vs. child age

Compared to children in the immobile age range (1–4 months), children in the mobile age range (5–14 months) had 0.17 log_10_ MPN higher *E*. *coli* counts on hands (Δlog_10_ = 0.17, 95% CI = 0.02, 0.32, *p* = 0.03). Within the immobile age group, there was no change in hand *E*. *coli* contamination with increasing month of age. Within the mobile group, *E*. *coli* on hands increased by 0.09 log_10_ MPN for each additional month of age (Δlog_10_ = 0.09, 95% CI = 0.01, 0.16, *p* = 0.03).

### Observed hand cleanliness

Among fingernails, 46% contained visible dirt particles, 31% appeared unclean and 23% appeared clean, while among finger pads and palms, 9–10% contained visible dirt particles, 16–17% appeared unclean and 73–75% were clean (Table 2). There was no difference in cleanliness indicators between the left and right hand.

**Table 2:** Δlog_10_ *E*. *coli* concentration on child hands associated with visible dirt particles and unclean appearance vs. clean appearance of fingernails, finger pads and palms^a^.

		log_10_ *E*. *coli*	Bivariate		Multivariable	
	N (%)	Mean (SD)	Δlog_10_ (95% CI)	*p*-value^b^	Δlog_10_ (95% CI)	*p*-value^b^
Dirt particles on fingernails	269 (46%)	0.92 (0.87)	0.10 (-0.06, 0.25)	0.23	0.03 (-0.13, 0.19)	0.72
Unclean fingernails	180 (31%)	0.84 (0.65)	0.01 (-0.16, 0.17)	0.94	-0.03 (-0.21, 0.14)	0.73
Clean fingernails	135 (23%)	0.82 (0.85)	Ref	—	—	—
Dirt particles on finger pads	54 (9%)	1.05 (1.06)	0.23 (-0.05, 0.51)	0.11	0.46 (0.05, 0.87)	0.03
Unclean finger pads	95 (16%)	0.98 (0.82)	0.16 (-0.02, 0,33)	0.07	0.16 (-0.27, 0.58)	0.47
Clean finger pads	435 (75%)	0.83 (0.75)	Ref	—	—	—
Dirt particles on palms	57 (10%)	1.01 (1.04)	0.18 (-0.09, 0.46)	0.19	-0.27 (-0.65, 0.11)	0.16
Unclean palms	99 (17%)	0.98 (0.83)	0.15 (-0.02, 0.33)	0.08	0.01 (-0.41, 0.43)	0.97
Clean palms	428 (73%)	0.83 (0.75)	Ref	—	—	—

SD: Standard deviation; CI: Confidence interval.

^a^Left and right hands combined into a single indicator for each hand part.

^b^*p*-value from generalized estimating equations (GEE) using indicator variables for “visible dirt particles” and “unclean appearance” against the reference category of “clean” for each hand part.

### *E*. *coli* on hands vs. observed hand cleanliness

The mean *E*. *coli* count on hands was 0.92 log_10_ MPN among hands where fingernails contained visible dirt particles, and 0.82–0.84 log_10_ MPN among hands where fingernails appeared unclean or clean (Table 2). The *E*. *coli* count on hands was 1.05 log_10_ MPN when finger pads contained visible dirt particles, 0.98 log_10_ MPN when finger pads appeared unclean and 0.83 log_10_ MPN when finger pads were clean (Table 2). Similarly, the mean *E*. *coli* count on hands was 1.01 log_10_ MPN when palms contained visible dirt particles, 0.98 log_10_ MPN when palms appeared unclean and 0.83 log_10_ MPN when palms were clean (Table 2). However, the differences between the cleanliness categories were not significant when comparing log_10_ *E*. *coli* counts for observations with visible dirt particles and unclean appearance against the reference category of clean for each hand part by GEE (all *p*-values >0.05, Table 2). In a multivariable GEE model with cleanliness indicators for all three hand parts, children with visible dirt particles on their finger pads had 0.46 log_10_ MPN higher *E*. *coli* on hands than those with clean finger pads (Δlog_10_ = 0.46, 95% CI = 0.05, 0.87, *p* = 0.03, Table 2).

Using our binary definition of “dirty” (visible dirt particles or unclean appearance) vs. “clean”, dirty nails detected binary *E*. *coli* presence with 81% sensitivity and 26% specificity, while dirtyfinger pads and palms detected *E*. *coli* presence with 29% sensitivity and 75–77% specificity. The composite binary indicator of dirty hands indicated *E*. *coli* presence with 82% sensitivity and 26% specificity.The PPV of observed dirtiness was 45–48% and the NPV was 59–65% for all four indicators (Table 3). Subgroup analyses by age demonstrated similar sensitivity, specificity, PPV and NPV for children aged 1–4 months vs. 5–14 months (Table 3).

**Table 3:** Specificity, sensitivity, positive and negative predictive values of observed child hand cleanliness against binary *E*. *coli* presence (<1 MPN vs. ≥1 MPN) by child age.

Hand indicators	Specificity, % (95% CI)	Sensitivity, % (95% CI)	PPV, % (95% CI)	NPV, % (95% CI)
**All children (1–14 months)**				
Fingernails	26 (22, 32)	81 (76, 86)	45 (40, 50)	65 (57, 73)
Finger pads	77 (72, 81)	29 (24, 35)	48 (40, 57)	59 (55, 64)
Palms	75 (70, 79)	29 (24, 35)	46 (38, 54)	59 (54, 63)
Overall hands ^a^	26 (22, 31)	82 (76, 86)	45 (40, 50)	65 (57, 73)
**Children 1–4 months**				
Fingernails	24 (18, 30)	81 (74, 87)	41 (36, 47)	65 (54, 76)
Finger pads	77 (71, 82)	22 (15, 29)	38 (28, 49)	60 (54, 65)
Palms	75 (69, 81)	22 (15, 29)	36 (26, 47)	59 (53, 65)
Overall hands ^a^	24 (18, 30)	81 (74, 87)	41 (36, 47)	65 (54, 76)
**Children 5–14 months**				
Fingernails	32 (23, 41)	81 (72, 88)	52 (44, 60)	64 (50, 77)
Finger pads	77 (68, 84)	40 (31, 51)	62 (49, 73)	59 (50, 67)
Palms	74 (65, 82)	40 (31, 51)	59 (46, 71)	58 (49, 66)
Overall hands ^a^	32 (23, 41)	82 (73, 89)	52 (44, 60)	65 (51, 78)

MPN: Most probable number; CI: Confidence interval; PPV: Positive predictive value; NPV: Negative predictive value.

^a^Composite indicator combining fingernails, finger pads and palms (i.e., any of these hand parts dirty).

When we repeated these analyses in low, medium and high *E*. *coli* contamination strata, the sensitivity of fingernails and hands overall did not vary between *E*. *coli* strata, while for finger pads and palms, the sensitivity increased from 26% to 41–44%with increasing level of *E*. *coli* contamination. For all four indicators, the PPV decreased while the NPVincreased with increasing level of *E*. *coli*. When hands carried 10–100 MPN or ≥100 MPN *E*. *coli* per two hands (vs. <1 MPN), the NPV was ≥90% for all four indicators (Table 4).

**Table 4:** Specificity, sensitivity, positive and negative predictive values of observed child hand cleanliness by *E*. *coli* contamination categories.

Hand indicators	*E*. *coli* level In hand rinse (MPN per 100 ml)	Number of samples in category	Number of dirty ^a^ observations	Number of clean observations	Specificity, % (95% CI)	Sensitivity, % (95% CI)	PPV, % (95% CI)	NPV, % (95% CI)
**Fingernails**	<1	333	245	88	26 (22, 32)			
	1–9	178	145	33		82 (75, 87)	37 (32, 42)	73 (64, 80)
	10–99	43	35	8		81 (67, 92)	13 (9, 17)	92 (84, 96)
	≥100	27	21	6		78 (58, 91)	8 (5, 12)	94 (87, 98)
	All≥1	248	201	47		81 (76, 86)	45 (40, 50)	65 (57, 73)
**Finger pads**	<1	333	77	256	77 (72, 81)			
	1–9	178	47	131		26 (20, 34)	38 (29, 47)	66 (61, 71)
	10–99	43	14	29		33 (19, 49)	15 (9, 25)	90 (86, 93)
	≥100	27	11	16		41 (22, 61)	13 (6, 21)	94 (91, 97)
	All≥1	248	72	176		29 (24, 35)	48 (40, 57)	59 (55, 64)
**Palms**	<1	333	84	249	75 (70, 79)			
	1–9	178	46	132		26 (20, 33)	35 (27, 44)	65 (60, 70)
	10–99	43	14	29		33 (19, 49)	14 (8, 23)	90 (85, 93)
	100	27	12	15		44 (26, 65)	13 (7, 21)	94 (91, 97)
	All≥1	248	72	176		29 (24, 35)	46 (38, 54)	59 (54, 63)
**Overall hands**^b^	<1	333	246	87	26 (22, 31)			
	1–9	178	145	33		82 (75, 87)	37 (32, 42)	73 (64, 80)
	10–99	43	35	8		81 (67, 92)	13 (9, 17)	92 (84, 96)
	≥100	27	22	5		82 (62, 94)	8 (5, 12)	95 (88, 98)
	All≥1	248	202	46		82 (76, 86)	45 (40, 50)	65 (57, 73)

MPN: Most probable number; CI: Confidence interval; PPV: Positive predictive value; NPV: Negative predictive value.

^a^Dirty defined as containing visible dirt particles or having unclean appearance.

^b^Composite indicator combining fingernails, finger pads and palms(i.e., any of these hand parts dirty).

Using the alternative definition for dirty (defined as only visible dirt particles) for a sensitivity analysis, dirty fingernails and hands detected *E*. *coli* presence with 47% sensitivity and 54–55% specificity, while dirty finger pads and palms detected *E*. *coli* presence with 10% sensitivity and 90–91% specificity. The PPV and NPV ranged from 42% to 58% (S1 Table).

## Discussion

Among our study population of very young children aged 1–14 months, 43% of the child hand samples were positive for *E*. *coli*. Children aged 5–14 months had significantly higher hand *E*. *coli* counts than children aged 1–4 months and, among the older age group, each additional month of age was associated with increasing hand contamination. Older children with increased mobility and motor skills are likely to have increased exposure to fecal sources in the domestic environment, are more likely to spend time on dirt floors or courtyards (rather than an elevated surface such as bed or mat) and also have the ability to pick up contaminated objects and/or feces. A longitudinal series of video observations among approximately 30 children aged 3–47 months in Bangladesh found no association between child age and the frequency of object touching but increasing age was associated with increased duration of touching objects [29]. The frequency of hand contact with soil was highest among children aged 6–11 months, and children who could crawl were more likely to touch animal feces compared to children who could not crawl yet, as well as compared to children who could walk already [29].

A previous analysis among our study population suggested increased hand *E*. *coli* contamination among children living in compounds that had domestic animals [12]. Rural Bangladeshi households usually raise livestock in close proximity to the family. In many cases, domestic animals are allowed to roam free within the compound and chickens are kept indoors within the dwelling at night. Since these practices are culturally ingrained, it may not befeasible to implement separation of animals from the household environment. Isolating animal feces from the domestic environment through prompt and hygienic disposal could reduce child hand contamination. Animal feces have various domestic uses in Bangladesh; cow dung is used as cooking fuel and chicken feces as fertilizer [30, 31]. Eliminating the presence or use of animal feces may not be practical but collecting and storing them for later domestic use at a location out of reach of children could reduce child fecal exposure and hand contamination. Moreover, open defecation in the household courtyard is common among young children in rural Bangladeshi households [32]. Tools to remove animal and child feces from the environment could reduce children’s hand contamination [33]. However, the parent WASH Benefits trial provided child potties and agricultural hoes for the removal of child and animal feces (as well as dual-pit latrines) to households in the sanitation arm and did not find any improvements in observed child hand cleanliness or hand *E*. *coli* counts compared to control households not receiving a sanitation intervention [34]. Hygienic play spaces to reduce child exposure to environmental fecal contamination might be analternative. There has been a growing interest over the past decade in interventions of protected play spaces for children for the prevention of accidents such as drowning, which could also reduce children’s exposure to animal feces [35, 36]. However, a recent study that delivered playpens for young children in Zimbabwe did not find a reduction in child diarrhea [37].

Our findings of poor associations between observed hand cleanliness and *E*. *coli* measured on hands are consistent with previous studies. In our analysis, *E*.*coli* concentrations were somewhat higher on hands that contained visible dirt particles than on those with unclean appearance, and somewhat higher on hands that appeared unclean compared to those that appeared clean, but the differences were not statistically significant. While children with visible dirt on their fingerpads had higher hand *E*. *coli* counts than children with clean finger pads in a model that included all hand cleanliness indicators, there were no associations between the other indicators and *E*. *coli* counts. The lack of significant associations could be due to the wide variability that has been documented for *E*. *coli* measurements on hands, making this a noisy outcome measurement [38, 39]. A study among farmers in Mexico also found no significant correlation between a visible hand cleanliness score based on observation of soil on hands and hand *E*. *coli* and total coliform concentrations while only a weak correlation was observed between the cleanliness score and *Enterococcus* counts on hands [20].This is in contrast with a previous study in Tanzania that found significantly higher *E*. *coli* and fecal *streptococci* counts on caregiver and child hands with vs. without visible dirt [4]. This could be because the Tanzania study had substantially higher hand *E*. *coli* levels and thus more power to detect differences, with mean *E*. *coli* counts on the order of 3 log_10_ colony forming units (CFU) compared to mean counts on the order of 1 log_10_ MPN/CFU in our study and the Mexico study.

When comparing binary hand cleanliness to binary *E*. *coli* presence, dirty fingernails detected the presence of *E*. *coli* presence with high sensitivity but poor specificity, while dirtyfinger pads and palms detected the presence of *E*. *coli* with high specificity but poor sensitivity, indicating that among children with *E*. *coli* on their hands the majority had dirty fingernails and among children with no *E*. *coli* on their hands the majority had clean finger pads and palms. The differences between the predictive performance of the fingernail vs. fingerpad/palm indicators suggest nuances in hand cleanliness across different parts of hands. It could be harder to remove dirt from fingernails than from finger pads and palms, and dirt under fingernails could provide an environment that better harbors microorganisms than the surfaces of finger pads and palms [6]. Overall, dirty hands predicted *E*. *coli* presence with a PPV less than chance (45–48%) while clean hands predicted *E*. *coli* absence with an NPV slightly better than chance (59–65%), altogether suggesting limited predictive performance. Nonetheless, clean hands predicted the absence of moderate/heavy *E*. *coli* contamination (≥10 MPN per two hands) with ≥90% NPV. This suggests that in situations where it is important to identify children without high levels of *E*. *coli* contamination of hands, clean hands can be used as an indicator with >90% accuracy.

One limitation is that the indicator organism in our study, *E*. *coli*, does not accurately reflect the presence of fecal contamination or enteric pathogens; therefore, detection of *E*. *coli* on hands as an outcome does not necessarily indicate a fecal-oral exposure through hands [40]. However, *E*. *coli* is a widely used fecal indicator. Its presence in drinking water has been associated with diarrhea [41], and our previous work showed that *E*. *coli* measured on child hands is associated with increased risk of diarrhea [25]. Additionally, observations of hand cleanliness is a subjective method as they depend on the enumerator’s judgment. Training and standardization is needed to ensure interrater reliability before they can be used reliably [42]. In an intervention trial, if the staff conducting the observations is not blinded, their judgment can be biased by their knowledge of participants’ treatment status [43]. However, our analysis was nested in the control arm of an intervention trial which received no handwashing materials or promotion. We therefore do not expect a systematic bias in our hand observations. Additionally, in studies with longitudinal visits, spot checks of hand cleanliness are vulnerable to participant reactivity. A previous study in India found improved visible hand cleanliness associated with repeat data collection rounds as participants improved their hygiene practices in anticipation of upcoming visits by study staff [44]. As our analysis is based on a one-time unannounced household visit,our hand observations should reflect the unaltered hygiene practices of the household. We conducted this study in rural communities from four districts which may not represent the broader rural community in Bangladesh or urban and peri-urban populations. Child hand contamination in urban Bangladesh may be different from rural Bangladesh as urban caregivers may have higher levels of education, animal ownership may be less prevalent and cement floors may be more common.

Our analysis demonstrated prevalent hand contamination with *E*. *coli* among young children in rural Bangladesh, with higher levels of contamination among children who can crawl and thus expose themselves to fecal sources in the domestic environment. Studies should assess if strategies to remove animal feces from the courtyard, provide designated hygienic play spaces and deliver targeted messaging to mothers to wipe or wash children’s hands after contact with animals and animal feces reduce child hand contamination. Visibly dirty hands were a poor predictor of *E*. *coli* presence on young children’s hands; alternative low-cost, field-based measures may be necessary to detect child hand contamination.

## Supporting information

S1 Table Sensitivity, specificity, positive and negative predictive values of observed child hand cleanliness by *E*. *coli* contamination categories using alternative definition for dirty vs. clean^a^. (DOC)
