# Supplementary material for: Microbiological contamination of young children’s hands in rural Bangladesh: Associations with child age and observed hand cleanliness as proxy
Source: PLoS One. 2019 Sep 10;14(9):e0222355. doi: 10.1371/journal.pone.0222355 (PMC6736272; doi:10.1371/journal.pone.0222355)
Supplement: S1 Table — (DOC) [file pone.0222355.s001.doc]

**Table S1: Sensitivity, specificity, positive and negative predictive values of observed child hand cleanliness by *E. coli* contamination categories using alternative definition for dirty vs. clean^a^**

| **Hand indicators** | ***E. coli* level**  **in hand rinse (MPN per 100 ml)** | **Number of samples in category** | **Number of dirty^a^ observations** | **Number of clean observations** | **Specificity,**  **% (95% CI)** | **Sensitivity,**  **% (95% CI)** | **PPV, % (95% CI)** | **NPV, % (95% CI)** |
| --- | --- | --- | --- | --- | --- | --- | --- | --- |
| **Fingernails** | <1  1-9  10-99  ≥100  All≥1 | 333  178  43  27  248 | 150  78  21  18  117 | 183  100  22  9  131 | 55 (49, 60) | 44 (36, 51)  49 (33, 65)  67 (46, 84)  47 (41, 54) | 34 (28, 41)  12 (8, 18)  11 (6, 16)  44 (38, 50) | 65 (59, 70)  89 (84, 93)  95 (91, 98)  58 (53, 64) |
| **Finger pads** | <1  1-9  10-99  ≥100  All≥1 | 333  178  43  27  248 | 25  15  4  6  25 | 223  163  39  21  223 | 91 (88, 94) | 8 (5, 14)  9 (3, 22)  22 (9, 42)  10 (7, 15) | 34 (21, 50)  12 (3, 28)  17 (7, 34)  46 (33, 60) | 65 (61, 69)  89 (85, 92)  94 (90, 96)  58 (53, 62) |
| **Palms** | <1  1-9  10-99  100  All≥1 | 333  178  43  27  248 | 33  14  4  6  24 | 300  164  39  21  224 | 90 (86, 93) | 8 (4, 13)  9 (3, 22)  22 (9, 42)  10 (6, 14) | 30 (17, 45)  11 (3, 25)  15 (6, 31)  42 (29, 56) | 65 (60, 69)  89 (85, 92)  94 (90, 96)  57 (53, 62) |
| **Overall hands^b^** | <1  1-9  10-99  ≥100  All≥1 | 333  178  43  27  248 | 154  78  21  18  117 | 179  100  22  9  131 | 54 (48, 59) | 44 (36, 51)  49 (33, 65)  67 (46, 84)  47 (41, 54) | 34 (28, 40)  12 (8, 18)  11 (6, 16)  43 (37, 49) | 64 (58, 70)  89 (84, 93)  95 (91, 98)  58 (52, 63) |

MPN: Most probable number; CI: Confidence interval; PPV: Positive predictive value; NPV: Negative predictive value.

^a^Dirty defined as containing visible dirt particles.

^b^Composite indicator combining fingernails, finger pads and palms(i.e., any of these hand parts dirty).
